# Constructing public–private partnerships to undermine the public interest: critical discourse analysis of *Working Together* published by the International Alliance for Responsible Drinking

**DOI:** 10.1186/s12992-023-01000-0

**Published:** 2023-12-16

**Authors:** Mary Madden, Andrew Bartlett, Jim McCambridge

**Affiliations:** Department of Health Sciences, Seebohm Rowntree Building, University of York, Heslington, York England

**Keywords:** Alcohol Industry, Commercial Determinants of Health, Corporate Social Responsibility, Global Health, Alcohol Policy, Public–Private Partnerships

## Abstract

**Background:**

The global burden of alcohol harm has increased and is forecast to grow further without effective policy implementation. Public–private partnerships aiming to address global health, and other societal challenges, are a burgeoning feature of neoliberal governance. Rhetorically distancing themselves from tobacco, the major alcohol companies are committed to tackling ‘harmful drinking’ and have created a distinct type of public relations organization for this purpose. The activities of such organizations are increasingly recognized as an impediment to the implementation of policies to reduce alcohol harm, including in low- and middle-income countries where markets are expanding.

**Methods:**

The approach of critical discourse analysis is used to examine the discursive tactics and strategies used in *Working Together*; a ‘toolkit’ published by the key global level alcohol industry public relations organization, the International Alliance for Responsible Drinking (IARD). This study considers how it works discursively to set the terms of, and overcome skepticism about partnerships, to define aims and position various actors by constructing their roles. The construction of prospective partners provides insights into the alcohol industry itself.

**Results:**

The toolkit operates as an ideological resource for forming public–private partnerships across the world based on the accumulated know-how of the major companies through IARD. This allows the largest alcohol companies to exercise leadership of the industry, while remaining off-stage. The toolkit relies on a form of rhetorical work which creates distance from obvious corporate interests and the harms caused to population health and society. This is accomplished by working *against* evidence-informed population level approaches, and thus avoiding policies that will make any significant difference to overall alcohol harm. Unspecific “complexity” affords opportunity for preferred types of “actions”, and “partnership” provides opportunity to gain credibility by association, further minimizing the likelihood of any material harm being reduced.

**Conclusions:**

The toolkit is designed to not only legitimate the inclusion of alcohol industry actors as initiating ‘partners’, but also assigns them roles as managers of a set of carefully constructed relationships. This vision of public–private partnership reproduces the hegemonic narrative that has successfully blocked policy advances for decades and led to growing alcohol harm globally.

**Supplementary Information:**

The online version contains supplementary material available at 10.1186/s12992-023-01000-0.

## Background

There is growing evidence in the literature on the commercial determinants of health (CDH) that the products and practices of transnational corporations are responsible for increasing rates of avoidable ill health, climate damage, and social and health inequity [1, 2]. As neoliberal governments promote public–private partnerships with large corporations to address health issues, this evidence has led to calls to recognize deleterious consequences, and to address the resulting harms [3, 4]. Such public–private partnerships often move responsibility for state regulation towards voluntary, privatized governance arrangements [5], diminishing a sense of the ‘public’ nature of public health and generating concerns about adverse impacts, particularly those stemming from unhealthy commodity sectors [1, 3, 6–9].

Consolidation in the alcohol sector means the global market is now dominated by a small number of producers, particularly of beer and spirits, that are targeting new markets in low and middle income countries [10]. Reversing the trajectory towards greater harm caused by alcohol consumption is a United Nations Sustainable Development Goal, and alcohol impacts many of the other goals [11]. There has been limited scrutiny, however, of public–private partnerships with alcohol companies to address alcohol harm [12].

During this period of consolidation, transnational companies have collaborated to form dedicated Corporate Social Responsibility (CSR) organizations, which are described in the sector as Social Aspects Organizations (SAOs) [13]. These organizations largely operate at the national level where they are highly involved in policy making [14]. The International Center for Alcohol Policies (ICAP) was founded in 1995 by a number of major alcohol producers at a formative moment, as hitherto largely nationally operating companies embraced new opportunities afforded by globalization [15]. The International Alliance for Responsible Drinking (IARD) was formed during 2014 as a result of the merger of its predecessor ICAP with a trade association, the Global Alcohol Producers Group (GAPG), with a mission to promote “responsible drinking” [16, 17]. ICAP has been more closely studied to date than IARD [15].

SAOs are often presented as independent organizations acting in the public interest in reducing alcohol harms [18–20]; ICAP made such claims, but IARD does not [21]. An examination of their origins and history based on internal documents identified that their core purposes were to counter major threats to business interests with public relations [18]. These organizations are thus primarily designed to advance the alcohol industry’s CSR and related policy influence goals [22–24]. They share some similarities with tobacco industry front organizations and more historical organizations formed by collaborating companies that were disbanded as a result of the Master Settlement Agreement with tobacco companies [18, 25], meaning these are now distinct in the alcohol industry [21]. The similarity with those disbanded organizations is in purporting to represent one agenda (public health) while serving the interests of an industry whose sponsorship and conflicting interests are downplayed, if not hidden altogether. IARD (and its predecessor ICAP) are unique in operating at the global (rather than national or regional) level, with the member companies being the largest global alcohol producers and appearing to play more strategic roles e.g., countering the World Health Organization.

SAOs are key vehicles for alcohol industry engagement in public–private partnerships, which have been proliferating in large neoliberal economies [26–29]. SAOs profess to have the same interests and goals as public health actors. The major alcohol companies, singly and together, argue that as part of responsible corporate ‘citizenship’, and despite producing a health harming, addictive product, they should be treated similarly to industries other than tobacco [30]. In so doing, they oppose the same set of policy measures that evidence has shown for decades are needed to reduce alcohol and tobacco harms: market interventions that increase price, reduce access, and restrict marketing [31–34]. There are striking similarities between tobacco and alcohol companies in their approaches to science, policy, and counterarguments to public health [35–38]. This is unsurprising as they have been in part co-developed [18, 25]. These stem also from strategic collaborations and through cross-ownership, meaning the two sectors have long been closely linked, and remain so today [25, 39–41].

As part of a research program investigating the alcohol industry, public health sciences and policy, we have studied closely a range of different industry actors operating in different policy and scientific contexts [12]. On the basis of this work, we identified IARD as particularly deserving of further scrutiny, due to the under-development of dedicated study of what appears to be the key actor globally [21]. In examining the IARD website and scoping out candidates for study we selected one particular document as warranting in-depth scrutiny, and so undertook the study reported here. This study is informed by the theoretical framework of critical discourse analysis (CDA) [42], which seeks to examine the power relations and ideologies embedded in concrete examples of discourse. Focusing on language use as a form of social practice, it offers new insights into rhetoric. The study aims specifically to explore the discursive tactics used in IARD’s *Working Together,* examining how it works to address and overcome skepticism about partnerships, to define the aims of partnerships and position various actors by constructing their roles, and so to function as a management guide to the formation of public private partnerships espousing alcohol harm reduction aims. In so doing, CDA—which has not yet been used much in the emerging CDH literature—may provide new insights into the nature of alcohol industry strategy, and how health interests may be advanced in a commercial determinants context.

## Method

### Data source

The object of study is the 2015 IARD toolkit *Working Together*, a short 24-page document (including covers) with contents organized as follows:

Introduction [3 pages] … Avoiding Possible Pitfalls of Working Together [3.5 pages] … Building Relationships Step By Step [1.5 pages] … Working With Different Stakeholders [3 pages] … Possible Areas of Collaboration [7 pages] … Appendix: Developing And [sic] MOU or MOA [3 pages] [43].

This toolkit is available as one of a suite of similar documents and policy reviews published on the IARD website [44]. The material in the "toolkits" section is diverse, with no set format or structure, covering topics ranging from self-regulation of advertising to drink driving. Some of the toolkits are ICAP documents predating the formation of IARD. Comparison between these toolkits and related documents is beyond the scope of this paper, which is focused in particular on the construction of ‘partnership’ in this toolkit, a standalone high-level guide to partnership selection and formation, “intended for those working in the beverage alcohol industry, including producers, retailers, trade associations, and social aspects organizations (SAOs)” [43] (p2). The inclusion of the latter is a rare or perhaps unique admission of the lack of independence from industry of SAOs.

The document has been selected to cast light on the partnership building strategy, which is a central, and perhaps even defining, aspect of the strategic approach to policy influence [30, 45]. The intended audience for the toolkit also makes it interesting to study. This is guidance published by the major global companies on how other actors in the alcohol industry may think about building partnerships at the national and sub-national levels. It is a guide for industry that is to a large degree *about* prospective civil society partners and how to work with them; civil society partners are not the audience.

### Approach

CDA focuses on the social and ideological underpinning of language practices (as action), with technical aspects of meaning making identified as evidence of these practices [46, 47]. The analytic premise is that the use of language reflects, reproduces, and can change power differentials between social groups. Here we use CDA to examine in depth structures of corporate policy discourse and its role in the hegemonic narrative that has successfully blocked alcohol policy advances for decades, in part by undermining and sidelining the population-level evidence-base [18]. We analyze the toolkit text critically, in playbook terms [48], as a form of CSR communication [49], and as a discursive obstacle to progressing evidence-informed policy measures because they negatively affect the alcohol industry’s conception of its interests. This methodological approach is in keeping with the text that is the object of study which itself stresses the importance of action, “sensitivity to local terminology” and the importance of, “choice of words … as these terms may connote different things to different people” [43](p3). Appreciation of the rhetorical sophistication, and power, of such content is integral to the analysis in this and other applications of CDA.

### Data analysis

We have adapted Fairclough’s five phase process approach to CDA for this context [47]:

1. Focusing on the semiotic aspects of alcohol industry interference in public health and policy initiatives.
2. Identifying and analysing the text of this toolkit and its location in a network of practices as an obstacle to addressing alcohol harm.
3. Considering how social networks and practices are organized to naturalise and perpetuate alcohol industry interference in public health and policy.
4. Identifying possible ways to contest the obstacles presented.
5. All while reflecting on our own positioning as publicly funded health researchers.

The toolkit is examined as a document that acts to reproduce the hegemonic ideas that legitimate the inclusion of alcohol industry actors as partners in initiatives to reduce harms from alcohol. That there are tensions or paradoxes in play between business interests and the espoused aims of those initiatives forms part of the analysis. The analytic focus is on ‘partnership’ and how this document functions as one component in a dynamic network of alcohol industry documents, actors and political strategies [50], to maintain and advance legitimation. This is in the face of evidence that alcohol industry CSR initiatives, despite their presentation, do not actually reduce harmful drinking, in part because they are not designed to do so [20].

For the detailed textual analysis, after initial familiarization with the document, its authors, and the website on which it was published, we summarized the explicit arguments found in the text; why “working together” is important, how this should be done, the opportunities and challenges of working with particular stakeholders, and exemplars of partnership. Open coding and categorization of the full text was then conducted line-by-line using NVivo 12 to identify underlying themes and semiotic aspects of problems, for which partnership is proposed as the solution. This showed how descriptive and rhetorical choices in the text strategically construct partnership. Frequency of key words was noted. Preliminary summaries, aspects of analyses, and the final analytic narrative were discussed in team meetings, and in text, as the analysis progressed. The Results section below presents findings on toolkit authorship and audience, how the text constructs the problems to be addressed and how it positions stakeholders and SAOs in providing putative solutions. Our findings are a critical reading against the hegemonic grain. To keep the analysis open to scrutiny we provide evidenced examples to show the reader how the data were interpreted and conclusions reached, and include reference to some other sources to critically discuss toolkit claims.

## Results

### Authorship and audience

The authorial subject position of the *Working Together* text is advisory. Authorship of the document is not specifically ascribed to individuals. Its production involves actors from ICAP/IARD—these were transitioning from one to the other at this time—their corporate backers, and the consulting firm LTG Associates Inc. A previous, undated version of the same toolkit was published by ICAP [51] and has undergone IARD rebranding. Toolkit provenance and presentation are examined in Additional file 1. The self-description of IARD in the copyright statement on the back of *Working Together* is:

a not-for-profit organization dedicated to addressing the global public health issue of harmful drinking. Our mission is to contribute to the reduction of harmful drinking and promote responsible drinking worldwide. This is a problem that requires new insights, urgent action, and open dialogue. Central to IARD’s work is our role as Secretariat of the Beer, Wine and Spirits Producers’ Commitments to Reduce Harmful Drinking.

As a “not-for-profit” organization, a term which carries connotations of selflessness and philanthropy, IARD operates as a gathering of executives of the largest companies, supported by professional staff [21]. Despite the well-established evidence-base on what works in policy [34], and the development of the WHO 2010 *Global Strategy to Reduce the Harmful Use of Alcohol* [52], the framing of the problem in the “mission” statement as “harmful drinking” is counterposed to “responsible drinking” as the solution. This is presented as requiring, “new insights, urgent action, and open dialogue”. The use of “new” and “urgent” implies that dialogue on “harmful drinking” is not currently open, that old insights and actions have not worked, and that ongoing or increasing industry involvement may contribute something new or different.

In terms of audience, while “primarily intended for industry members”, prominently including SAOs, the document is described as, “a generic tool that can be applied in any number of circumstances by any group looking to forge alliances and partnerships” (p2). This implies that issues specific to alcohol, its material properties and the relationship of industry to the harms produced by alcohol may be of little substance. Rather, this is an industry like any other, producing an ‘ordinary’ commodity [34], and so the toolkit could be of broad relevance to members of any industry seeking to work with non-industry actors. Key parts of the succeeding content, however, are very specific to alcohol and alcohol policy issues, such as how the public health community and its evidence-base are to be regarded (see below).

### Purpose, problem definition and positioning SAOs in solutions.

The text presents five “models” of working together, referencing a chapter titled *Working Together* in the ICAP edited collection on *Working Together to Reduce Harmful Drinking* [53], a book based on industry submissions to the WHO 2010 *Global Strategy* consultation*.* These list the lowest preferred level of industry involvement with stakeholders as, “providing resources”, and the highest as “partnership”. In ascending order, the others are, “sharing best practice”, “developing and implementing codes of practice” and “developing and implementing programs” (p4). The “common goal” (p4) of those working together is to address a global public health problem framed as an issue of “harmful drinking” (this term occurs 14 times, “harmful use of alcohol” five times and “alcohol-related harm” four times). The importance of partnership is made clear: “Real partnerships represent the highest level of working together and can be transformational” (p4). It is nowhere made clear, however, what can be transformed, or for whom. The goal is to achieve, “some form of collaboration in which stakeholders work toward a common goal within a co-created organizational structure” (p3), thus subsuming a range of outcomes that may otherwise be articulated as effects on rates of alcohol harm. Perhaps unsurprisingly, the evidence indicates a close relationship between the overall level of alcohol consumption in a population, and all forms of harm in society, so a reduction in drinking per se can be expected to lead to a reduction in social and health harms [34, 54].

Partnership is one means of securing a role in problem definition, as well as in the responses that result, and this is promoted here as a way to do the former and shape the latter. “Real” partnerships involve industry working together with collaborators to, “define problems and work towards solutions” (p4), with the caveat that this, “might not necessarily cure social, environmental, or public health problems entirely,” but “can play an important role in reducing the harm caused by these problems” (p5). In outlining three, “basic steps to build a balanced working relationship” the text locates the toolkit user (an industry actor) as the active, initiating agent in ‘co-creation’ allocating the verbs to them: “Outline the goals … Assign someone … Determine the parameters …” (p6). A sample memorandum of understanding (MOU/MOA) included as an appendix follows a precedent set by the Portman Group in the UK [55], and this is included along with other materials that have proven useful previously. The WHO is invoked to provide legitimation for the idea that the industry can be a partner:

... under: (1) The recommendations of the World Health Organization’s Global Strategy to Reduce Harmful Use of Alcohol, which provides that the alcohol industry may enter into joint projects with nonprofit, research, or public organizations to address the harmful use of alcohol (p21).

The space for such rhetorical devices to be deployed was made possible by weaknesses in the original 2010 version of the *Global Strategy* which states, “economic operators … important players in their role as developers, producers, distributors, marketers and sellers of alcoholic beverages … are especially encouraged to consider effective ways to prevent and reduce harmful use of alcohol within their core roles” (45d) [52]. Because such weaknesses facilitated policy interference, a stronger proposal for implementation was later adopted in 2022 [56, 57]. The WHO 2010 *Global Strategy* is thus presented in this toolkit, via a series of industry self-referencing interpretations, as offering not just, “clear opportunities for working together” (p13), but the authority for industry involvement. Contributions that industry might make, according to the Toolkit rather than WHO, are listed as follows:

- building relationships among industry members, government bodies, community organizations, and others;
- implementing programs at local or national levels;
- encouraging and supporting initiatives where they are absent or weak;
- implementing sustainable and culturally appropriate programs;
- evaluating initiative processes, outcomes, and impact (p2).

### Putative solutions and their evaluation

The toolkit states that “the alcohol industry has focused its attention on targeted approaches” and provides 29 existing examples of its work to promote these such as, “education, drink-drive initiatives, interventions for problem drinkers, creating safe drinking environments, and the self-regulation of marketing and advertising” (p15). Exemplars are presented as foundations on which to build, showing the opportunities “**to take concrete action**”, by “targeting specific at risk groups in particular cultural contexts” (p19 bold in original). Examples of industry involvement in reducing alcohol-related harm, or avoiding whole population measures, are drawn from across the world, most frequently from South Africa (n = 5). Substantive supporting evidence that these make any difference to rates of alcohol harms is entirely absent, notwithstanding the evaluation contribution suggested above, or any possible doubt about the technical competence to do work of this nature (or indeed the other contributions promoted). Some exemplars are about facets of the industry acting on itself, while the rest are clearly outside the expertise of alcohol producers, marketers and retailers, but do find indirect roles for industry actors in areas where they may have some standing, resources or access. Much of this is focused on promoting “awareness” among health professionals and consumers with no clarity on whether and how proposed changes in “awareness” lead to changes in drinking, much less how any changes in drinking have an appreciable impact on harm. For example, the presentation of market research evidence used to support an “evaluation” of a “campaign” with health professionals reported outcomes in the following terms:

An evaluation of this campaign, conducted in 2007, found that one third of the medical professionals interviewed believed that they had reduced problems associated with drinking during pregnancy and two thirds reported that they were now more confident talking to their patients about alcohol (p13).

One of three other references to “evaluation” in the exemplars refers to IARD as a provider of evaluation in its role of capacity building to address drink driving in six targeted countries (p17). Web references to reports of “an independent evaluation” of a community alcohol partnership on underage drinking by a trading standards organization and to an evaluation of a scheme to promote responsible operation of licensed premises are no longer working. “Evaluation reports” for the latter are said to find reductions in alcohol-related crime and savings to the health service as well as, notably, “an increase in trade of 28%" (p15), with the caveat, “it is not possible to attribute all of these findings to BBN [the Best Bar None intervention] exclusively” (p15).

Where scientific studies have been undertaken of the data behind the claims made in such evaluations, the claims are usually found to be hollow [58, 59]. Industry self-regulation exemplars include reference to the code of practice provided by the Portman Group, “another social responsibility body for alcohol producers” to supplement, “already stringent requirements” (p18). Portman has been found to strategically mix accurate, misleading and distracting information in its messaging [60].

Also included is IARD itself as an example of working with transnational bodies and local partners in low- and middle-income countries, “to develop and implement industry-specific responsible marketing” (p18), in places with currently fewer such “stringent” requirements (p18). The evidence on marketing supports comprehensive regulatory restrictions, not self-regulation [34]. None of the examples featured include action on the availability of alcohol (other than to children, p16), or pricing policies, for which evidence is strongest [34], but which are also inimical to industry interests. These focus instead on promoting “responsible” consumption via awareness, screening, education, training and safety programs, upholding industry self-regulation and taking action on “noncommercial alcohol”:

.. Industry statistics suggest that up to 50% of the world’s supply of beverage alcohol is illicit or informal … Providing goods and services that meet the needs of low-income consumers while employing the local population is one way in which large businesses can help reduce health risks and improve the economy (p19).

Action against illicit alcohol is presented as warranting securing tax breaks for industry and providing increased employment through developing cheap brands for sale in Kenya, Uganda, and Zambia (p19), thereby expanding markets.

### Identifying opportunities and positioning stakeholders

“Working together” is identified as, “important for the alcohol industry” for three reasons, all opportunistic in nature (pp2-3):

1. Th﻿e opportunity for multisectoral engagement is a growing and exploitable trend: “strategic alliances … are a growing trend in social and policy development” (p2).
2. Unspecific “complexity” affords opportunity for industry input: “no one sector by itself can address the complexities surrounding many issues” (p2).
3. An opportunity to gain credibility by association: “[a]lthough alcohol industry members are committed to reducing alcohol-related harm and promoting responsible drinking, they may lack the necessary expertise or the credibility to do so effectively” (p3).

The note on expertise is hedged by credibility considerations, and in all cases the specific nature of the opportunity available, or the benefit to industry, is left open. Opportunity is therefore afforded by trends in governance and “complexity” [61], with partnership presented as a means of broaching an acknowledged deficit in industry “expertise” and, importantly, “credibility” for the proposed “contributions” previously described: “Therefore, working with others better positioned to engage in these areas is valuable and important for industry members” (p2). This admission of a lack of necessary expertise or credibility, and the opportunity to gain these by association, indicates that the alcohol industry is not acting within its “core roles” [52] when it seeks to enter “into joint projects with nonprofit, research, or public organizations to address the harmful use of alcohol” (p21).

Building relationships is the first identified contribution to be made by industry in the document’s list of such candidates (p2), and the existing literature identifies the centrality of long term relationship building to the political strategies of the major alcohol companies [30]. The toolkit focuses on developing relationships necessary for working together and stresses the importance of awareness of the “cultural, political, and social context” (p3). Given the global targeting of the toolkit, sensitivity to “cultural issues” is stressed and working with a good “cultural translator” is recommended where alcohol consumption is not the cultural norm, for example, “when trying to address alcohol misuse in a ‘non-drinking’ culture, or when dealing with issues of fetal alcohol syndrome in a culture where ‘women do not drink’” (p7). There are 13 references to “communication” in the document and discussing and agreeing on, “a communication strategy”, is said to be important given those that industry seeks to work with, “may differ in their methodological approaches” (p6). The purposes being served by the recommended relationship building or communication are vaguely presented, and as such the foregrounding of these processes implies these are ends in themselves rather than any kind of means to an end.

“Working together” itself can be defined strategically in linguistic terms, “which best suit the parties involved”:

… an alliance, coalition, coordination, cooperation, or partnership … The choice of words is important, as these terms may connote different things to different people. Some may be value-laden, others more neutral (p3).

This pragmatic approach advocates using whatever works to make, “stakeholders feel valued and comfortable” (p3), without—as might perhaps be expected in a business context—attending to the detail of the different legal roles and responsibilities implied by each of the potential “co-created organizational structures” (p3). In contrast to this ambiguity, clearly defining roles for “stakeholders” is presented as “essential” (p3), after having inserted the industry actor as a stakeholder themselves without a specified role other than the implied role of director of the ensemble. There are 51 references to “stakeholder(s)” in the document, with a definition provided in a text box:

Stakeholder: an individual or group with an interest in the success of an organization in delivering intended results and maintaining the viability of the organization’s products and services (p3).

That a stakeholder must have an interest in, “maintaining the viability of the organization’s products and services”, indicates that stakeholders are not therefore defined by their ‘stake’ in a *problem*. They are defined instead through a commitment to a particular form of outcome for the organization itself, and not necessarily its declared ‘social aspects’ aims in relation to the problem. Indeed, candidate stakeholders primarily interested in evidence-informed solutions are inherently problematic from the outset, as they privilege solutions to problems over organizational needs. For these potential stakeholders to become ‘partners’, some level of acceptance of industry framings of the nature of the enterprise, and the involvement of the industry within it, is required. Later in the text, the toolkit acknowledges that, “some prospective stakeholders may hesitate to work with the alcohol industry” (p9).

Potential “collaborators” with industry stakeholders are listed as follows (p10-12):

Government Sectors, Public Health, Nongovernmental Organizations (NGOs), Community-Based Organizations (CBOs), Academia, Media, Public Interest Groups, Professional Associations, and Consumer Organizations.

Specifc opportunities and challenges are said to be afforded by each group (see Table 1). This is perhaps where the toolkit comes closest to functioning as an operating manual, containing material on who to work with, why, and for which purposes. “Opportunity” is focused on the potential to enhance industry credibility and extend its influence over and through collaborators, as identified when the toolkit was created (pp10-12):

**Table 1:** IARD *Working Together* toolkit: opportunities afforded by potential partners

*Sector*	*Opportunity afforded*
Government Sectors	Work with providers of the regulatory framework to address, “harmful drinking” and also, “promote the benefits of alcohol as an economic resource” with e.g., “agriculture, trade, transportation, or tourism”
Public health bodies	“… have a powerful voice in matters of health policy … an opportunity to demonstrate what types of approaches to reduce harmful drinking work in different economic, social, and cultural settings”
Nongovernmental Organisations (NGOs)	“… can play a substantial role in advocacy and shaping policy… working with targeted populations, possible access to community gatekeepers … may serve as a ‘local’ stakeholder to nonlocal industry organizations, or may already have projects in place that need support”. Experience of planning and implementation “on the ground”. “They may also have extensive networks that can be put to good use.”
Community-based organizations (CBOs)	Community ties and access to gatekeepers to help target particular neighbourhoods or populations
Academia	Work with those who, “provide recommendations based on best practices and … evidence-based alcohol advice … well respected as experts … strive for transparency and high ethical standards.”
Media	“The media may help the industry build trust … can be a powerful ally in educating the public by bringing positive attention to the various initiatives of the alcohol industry.”
Public interest groups	“… increasingly aware of some of the unintended, negative consequences of whole-population approaches to alcohol control favored by an important sector of the public health community, and might be open to innovative or promising interventions targeting vulnerable groups.”
Professional associations	“… are recognizing that interventions targeting at-risk populations are necessary to deal with alcohol-related harm … the scientific evidence that they deal with contains fewer gray areas … interested in providing accurate and balanced information to their audiences and constituencies.”
Consumer organizations	“… take into account what the general population actually wants, without necessarily adopting an advocacy position. They are also most interested in freedom of choice for their constituents”

Opportunities are presented in terms of potential receptivity of partners to industry ideas and lack of hostility to industry interests, perhaps most notably with regard to “the unintended, negative consequences of whole-population approaches” (p11), and the scope for credibility enhancement. This offers a high level guide to making assessments of the suitability of prospective partners. Most receptive are those open to “balanced” information, especially consumer organizations which, “take into account what the general population actually wants” and are in sympathy with industry’s own advocacy of “freedom of choice” in the market (p12). However, these are, “few and not well organized in the developing world”, and this can be overcome by, “[w]orking together with different industries” to “help to raise visibility and strengthen emergent consumer organizations” (p12). The global and historical contexts matter in relation to all prospective partners. The toolkit asserts, “basic principles”, examples of “good practice” and warnings about how to avoid possible pitfalls, “through a better understanding of some of the ideological, methodological, and cultural issues that may arise” (p2). The issues in play are presented as high stakes and requiring deft handling to minimize threats to industry interests, here constructed as misunderstandings of industry motives.

### Overcoming the key challenge

The toolkit repeatedly invokes the importance of achieving “balance” (used 8 times) and “trust” (used 13 times). Partnerships require, “work to maintain a fair balance of power” (p5), defined as: “parity or stability between competing forces” (p6). Meeting the challenge of, potential, “ideological, methodological, and cultural” tensions (p2) between such “competing forces” is reduced to a single point of conflict; managing the (mis)perception of the interests of the alcohol industry on the part of potential partners. This goes to the heart of the exercise in legitimation; anyone who disagrees with this vision of partnership with the industry has an issue. For example, some in the government sector, “… may question the motives of the alcohol industry in addressing harmful drinking, and thus may be unwilling to engage with industry” (p10). It is notable that the toolkit suggests that the most challenging groups for industry to work with are public health bodies, specifically as a result of their support for evidence-informed, whole-population measures over the preferences and material interests of industry:

Traditionally there has been some distrust and tension between the public health community and the alcohol industry. In particular, some who support whole-population policies to reduce total per capita consumption and exclude targeted interventions - by increasing taxes, restricting licenses and availability, adding or expanding warning labels, increasing the legal age for purchase or consumption, or banning advertising - may have a position at odds with that of industry members … may be suspicious of motives and believe that public health goals and profit making are mutually incompatible (p10).

The public health evidence-base on interventions that may effectively reduce alcohol harm becomes merely a position. This brings to the fore the role of IARD (and its predecessor ICAP) as a form of corporate impact investment in “not-for-profit” social responsibility branding, aimed at keeping whole population ideas and evidence on alcohol harm and policy responses outside the frame of partnership. Their inclusion would necessarily position public health - or more specifically meaningful progress towards attainment of public health goals- and the appearance of activity in the name of public health, which makes no dent on capacity for profit making, as conflicting goals. The idea that the material interests of the alcohol industry might actually be opposed to the interests of reducing alcohol harm is repressed in the text except as a “suspicious” misperception, which is to be corrected by more contact with industry in partnership working.

“Transparency” (see below) will allow suspicious potential partners to see that they were in error. Suspicion of motives because the industry is, “profit making” (or being “’for profit’” (p7) placed in quotation marks in the text as if this were a problematic term) is also attributed to NGOs and CBOs, organizations which, “generally need resources but may even have clear rules against working with industry for ideological reasons” (p10). Suspicions, “motives” and “beliefs” are attributed non-dialogically to a mute opposition presented as intrinsically against profit making. This adroitly bypasses consideration of the nature of the conflicting interests, and clearly locates the problem in abstract ideas amenable to correction by building closer relationships with industry actors.

“Skepticism” about working with the alcohol industry “may also be found” among professional associations and public interest groups who, “tend to align with the messages of the public health community” (p11). Ironically, on the one hand there is a framing that suggests that complex social and health problems globally are best defined and addressed in partnership with corporations that lack expertise in the problems caused by their own actions and lack interest in evidence-informed solutions. On the other hand, those opposing industry framings are positioned as doing so for “belief” based and “ideological” reasons rather than evidence-informed reasons. Indeed, the suggestion that public health bodies “traditionally” distrust the alcohol industry can be read as implying habitual conformity and resistance to innovation, and that partnership with industry opens up the possibility for change among those inclined to reject tradition. The toolkit user is thus prepared to embark on the enterprise of partnership building with this particular version of the conflict to be anticipated and managed.

The text depoliticizes policy, implicitly denying that industry actors have policy goals to advance their own economic and political interests. It renders partnership problems as attitudinal phenomena of opponents who may be better dealt with by exclusion from partnerships. Academia is another sphere in which there may be, “a perceived conflict of interest, though the Dublin Principles may help to mediate such fears” (p11). This is one of only two explicit references to “conflict of interest” (p11) and “conflicting interest” (p5) in the text. The Dublin Principles of Cooperation were an ICAP initiative seeking to promote partnerships between the research community and industry actors [62]. The toolkit constructs those academics willing to enter arrangements on industry terms as shy of being seen to be political or of engaging in “advocacy”:

It is also important to understand that most academics are not advocates and that they may not be interested in collaborative efforts where advocacy or political debate plays a role (p11).

This approach has obvious implications for academics who may hold views about industry, or indeed have studied industry activity or alcohol policy, and how they might be regarded. In addition to the Dublin Principles, the toolkit explicitly suggests that industry engages in a practice that has long been used by harmful commodity industry PR groups to obfuscate the role of industry, keeping themselves out of the story, and enabling those participating in this way to rationalize that they are independent:

There are ways to provide funding in a hands-off way—for example, through third parties—and thereby maintain distance and independence (p11).

Working with the media presents the challenge of competition for attention and the danger that negative, “sensationalist” stories might be preferred because they sell well but might make the industry look bad (p11). Success can also bring difficulties if such coverage is, “perceived by some as thinly veiled marketing and promotion” (p11). CSR branding that looks like naked PR can undermine the normative extension of the corporate role:

Look beyond the opportunity for better PR and focus on sustaining corporate social responsibility (CSR). CSR means that companies should go beyond manufacturing good products and pay attention to employees and employee treatment, the environment, the local community, and the wider society and culture in which the company operates (p9).

### Transparency

Notwithstanding the guidance on discretion in funding, there are 12 references to “transparency” in the toolkit, mostly linked to financial ties. Ensuring transparency in a relationship is said to build trust and facilitate communication among stakeholders. If conflicts are not of a material character, but are merely issues of perception, then ‘transparency’ is a relatively straightforward means of dealing with these conflicts, particularly so if the most troublesome (ideologically incommensurable) parties have been excluded. Through disclosure and acceptance, misperceptions will evaporate:

Most importantly, transparency involves being open and clear about any perceived competing or conflicting interest that may play a role in outcomes. In partnerships involving industry stakeholders, the most common issue where transparency is needed is around financial ties and their perceived impact on the final product (p5).

Outcomes are here defined in terms of perceptions. It is not clear in the toolkit whether the “final product” is the partnership itself or the work the partnership does, and how this relates to the “outcomes”. Conflicts between partners in this text can never really be about genuinely competing or conflicting interests, because these are framed as “perceived” and not about real interests:

As long as the stakeholders involved are open and clear about their positions and they maintain lines of communication and compromise, they should be able to weather the challenges they will inevitably face. Trust, transparency, and balance are the key components in building and maintaining a strong, rewarding, and successful working relationship (p8).

This self-reinforcing logic means that conflicts of interest become the epiphenomena of contentless ideological conflict. Conflicts of interest are thus carefully designed out of this model of partnership building. A trusting recognition and acceptance of differences in “perception” is sufficient. “Working together” relies on building an impression of, “parity or stability between competing forces” (p6):

This means sharing information and resources, as well as risks and rewards. It also requires mutual respect from the outset, as well as the willingness to be inclusive and to contribute equally to the process. Above all, working together must rely on transparency with regard to goals and also any potential competing interests among stakeholders (p3).

Despite the importance of “competing interests” conveyed by the phrase, “[a]bove all”, the actual interests of industry actors recede as important in this toolkit where there is a particular form of transparency “with regard to goals”. “Working together” is said to “thrive” on compromise, which means, “both parties must be willing to concede something in pursuit of the greater good” (p7). Actual solutions in the form of measures that will make some difference to alcohol harm, about which there can be confidence based on existing evidence of effectiveness, do not appear in the toolkit. From the perspective of the industry, with an interest in maintaining and developing its market, working together becomes an end in itself. It may be a “challenge” to reconcile interests in evidence-informed approaches to alcohol with a “balanced” harm reduction mission that dismisses population level approaches out of hand (p10), promotes the benefits of alcohol as an economic resource (p10), focuses only on the positives in evaluating its own approaches (pp13-15) and, despite advocating transparency in partnerships, without any sense of irony guards against “adding or expanding warning labels” for consumers (p10). Hence perhaps, the narrow co-opting definition of potential stakeholders as those with “an interest in […] the viability of the organization’s products and services” (p3).

## Discussion

The results of this study show how this IARD toolkit operates as an ideological resource for forming public–private partnerships, allowing the largest corporations globally to exercise leadership of the industry while remaining off-stage. The toolkit relies on a form of rhetorical work which creates distance from obvious corporate interests and the harms caused to population health and society. This is accomplished by working against evidence-informed population level approaches, and thus avoiding policies that will make any significant difference to overall alcohol harm. Unspecific “complexity” affords opportunity for preferred types of “actions”, and “partnership” provides opportunity to gain credibility by association, further minimizing the likelihood of any material harm being reduced. We now reflect on the key issues raised by this analysis and their implications, before considering its contribution to the literature and research implications.

Being part of the solution rather than the problem has been the key long term alcohol industry strategy since the end of national prohibition in the United States of America [18]. This meant firstly that the public relations problem had to be defined as alcoholism or alcohol abuse and not anything inherently to do with alcohol or the industry that produces it. Secondly, for decades resources were directed into education and research in an approach developed in parallel with the tobacco industry at around the same time and involving the same public relations company [18]. In this context *Working Together* can be seen as representing an iteration of this long-term approach, promoted by the major alcohol companies for other actors in the industry across the world. Other sectors have adopted similar approaches to those developed by alcohol and tobacco [63–65] in attempting to become part of the solution, for example the food industry in relation to obesity [66].

Given the ubiquity of commercial alcohol availability and marketing in high-income consumer economies, extending reach in low- and middle-income countries, and the normalization of alcohol harm, whole population ways of understanding alcohol harm and evidence-based public health policy measures are counter-hegemonic. They are disruptive of what has been the status quo, and so face obstacles in displacing ‘common-sense’ approaches that are in line with the interests of powerful commercial actors [67], about which there is silence in discussions of roles and responsibilities for harm. This is particularly the case in countries where neoliberal ideas are dominant, though even in such contexts important advances in alcohol policy in the interests of public health can be made [68–70].

Far from “open dialogue” (back cover), the IARD *Working Together* toolkit attributes fixed ideological positions to others in need of correction, shaping partnership discourse flexibly to its own ends. The industry actor is to exercise partnership initiation and managerial functions, with crucial attention given to who is included and on what terms. Transparency is invoked as a means to create trust, but a weak form of disclosure is advanced that preserves space for obfuscation and distancing techniques (such as presenting an industry body as “not-for-profit” and providing funding through third parties). This is some way from the “complete transparency” regarding CSR “strategy, motives, objectives and measurement tools” that Dunfee claims, “firms acting consistently with social expectations, norms, and laws should be happy to disclose … because this should enhance their reputations” [71]. In the medical arena there is growing concern that such transparency (declaring conflicts of interest) is necessary but not sufficient for managing the distortions across research, education, practice and policy resulting from commercial influences on health, and independence should be the goal [72].

Implicit throughout the toolkit, in both the hypotheticals and the examples of successful ‘partnerships’, is that alcohol industry actors have something to contribute to these partnerships. It is nowhere specified exactly what this is. While there is the concession that alcohol industry actors, “may lack the necessary expertise or the credibility to do so effectively” (p6), they nevertheless expect a seat at the table to play an active role in shaping, implementing, and evaluating “sustainable and culturally appropriate programs” to deal with alcohol harm (p2). They claim to do so as stakeholders, yet industry actors’ principal roles are in the constitution of the harm [73]. They have a stake in the problem, in that their profit-seeking produces harm, and as such they have an interest, but not expertise. Moreover, the claimed contributions afford industry actors further power to defend their reputations and act against the regulatory measures that are known to have capacity to reduce harm from their products. In this sense, partnerships with major alcohol companies are dysfunctional because the consequences are harm promotion not harm reduction [74].

What the toolkit offers is consideration of, “different types of stakeholders with which industry members might engage”, not in order to understand and ameliorate harm as those stakeholders define it, but to understand the “opportunities and challenges posed by various partnerships” (p2) in engaging with industry’s own particularly narrow and self-serving definition of the harm caused by alcohol. While there is acknowledgement that harm occurs, and that this is, “a global public health issue”, the problem to be addressed is framed narrowly. Moreover, the industry is presented as part of the solution, the boundaries of which are constrained by the very name of the organization, “International Alliance *for* Responsible Drinking”. The promotion of drinking worldwide, with the caveat that consumers do this ‘responsibly’, is long-standing [16, 18]. The preferred terms in the toolkit text similarly relocate responsibility for alcohol harm solely in the agency of those drinking, rather than in the properties of the legal product itself, or by extension, its producers and retailers. In a discourse familiar from other harmful commodity industries, IARD—as the “Secretariat of the Beer, Wine and Spirits Producers’ Commitments to Reduce Harmful Drinking”—imply that alcohol is an ordinary commodity and that it is *misuse* that creates harm, positioning producers as the responsible actors who can advise policy makers, retailers, and the end users on how to act responsibly [34, 75, 76].

There is plasticity in the definition of stakeholder and the hands depicted on the cover of the toolkit can perhaps be seen as representing the spin of its circular reasoning. Potential collaborators are invited to engage in a process of corporate neoliberal responsibilization [77], which transfers *responsibility* for alcohol harm to faulty consumers [78], while at the same time presenting industry as a socially *responsible* sector willing to tackle complex issues, even to the point of working with organizations with supposedly challenging ideological orientations. There is also a form of circularity in its advocacy of partnership with responsibilizing organizations which are promoting ineffective strategies as a solution to alcohol harm, thereby implicitly further increasing the supposed “complexity” that affords opportunity for such industry input. Any humour to be found in pointing out such twisted logic is countered by the deadly consequences. The user would not know from reading the toolkit that the deaths of three million people every year are attributed to alcohol consumption [79], with countless more lives ruined, and that constructing partnerships in this manner will make the situation worse not better [74].

That “working together” can be defined strategically in terms, “which best suit the parties involved” resonates with the critique of strategic ambiguity of “partnership” in Global Health Partnerships identified by Taylor (2018), not as a matter of miscommunication but as deliberately, “ripe for mutual misunderstanding”:

… the capacity of the word ‘partner’ (or by extension ‘partnership’) to encompass such divergent understandings, and in effect to facilitate mutual misunderstandings, is arguably precisely why it can bring together assemblages of people and organizations across great distances and steep gradients of inequality [80].

The capacity of this “toolkit” to function as a practical guide to the day-to-day work involved in building partnership for industry actors is secondary. The primary purpose served is as an ideological resource to equip actors across the industry and across the world to prevent effective actions on alcohol being taken; the toolkit is designed for co-optation of actors who may be useful. Within the text, collaborators are to be acted *upon* rather than *with*. Industry reliance on acquiring credibility (cultural capital) and authoritative legitimacy from potential partners carries with it the means of undermining public confidence in the trustworthiness of those partners and in their expertise. Sismondo describes this, in relation to the pharmaceutical industry, as a form of “grafting”, where collaborators with industry can find themselves co-opted for interests outside and other to their stated goals [81].

IARD is grossly understudied given its significance to global health and we suggest this study of IARD permits greater understanding of the CSR strategies and practices of the major alcohol companies. The poverty of the actual content of this toolkit does not detract from the insight this document provides into the power of the overarching approach; this is one component in a wider set of mutually reinforcing political strategies to maintain the status quo. We have not examined how the document is used, which would require an altogether different, and more complex study. Instead, it has been analysed as a publicly accessible document from ‘inside’ the alcohol industry. In-depth engagement with this one text does not fully address its relationship to other toolkits and other materials produced by these actors, or other materials produced by their member companies. We encourage such studies. We have undertaken this study from a health perspective. We are also informed by prior studies of the alcohol industry, a literature to which we have contributed. This confers advantages of many kinds and also implies some potential for the careful empirical work on the text to be influenced by our approach and prior work. We invite the reader to bear such considerations in mind. In taking further the suggestions for further analyses made here, we invite social scientists with different backgrounds to make contributions.

Here, CDA has afforded insights into discursive dynamics and how this particular document ‘works’, including in the exercise of rhetorical power and its self-generating tensions and limits. Any one analytic approach may be critiqued, and we invite further studies of this document that employ other analytic techniques. Furthermore, textual analysis of such documents needs to be augmented by studies of their use in action in different contexts to build partnerships, advance CSR goals, influence policy, and exercise political power more broadly. This includes further study of how partnerships are actually formed, particularly in national policy making, in ways that follow or depart from this guide. Careful attention to organizations such as IARD also has implications for study of these issues in other corporate sectors. Comparative studies that examine how CSR is strategized and operationalized by transnational corporations in relation to public policies across different sectors will form part of the emerging commercial determinants of health research agenda [1, 8].

## Conclusion

The IARD *Working Together* toolkit operates as a resource for forming public–private partnerships based on the accumulated know-how of the largest alcohol companies globally, while they remain off-stage. It attempts to legitimate the inclusion of alcohol industry actors as ‘partners’ while reproducing the hegemonic narrative that has successfully blocked alcohol policy advances for decades. This approach was developed over many decades alongside the tobacco industry. As this approach appears to have been largely successful to date in alcohol policy, emulation of this by other health harming industries may be expected. There is a need for a closer examination of the discourse and tools of public–private partnership working across unhealthy commodity industries, and examination of their effects on policy decision-making. The need to protect progress towards public health goals from unhealthy corporate interests is not going to disappear, especially when it is being made to seem that vested material interests can vanish.

### Supplementary Information

**Additional file 1:** **Fig. S1.** Toolkit cover from *International Alliance for Responsible Drinking *[1] and earlier version from *International Centre for Alcohol Policies *[2]. **Fig. S2.** Recurring image in ICAP Toolkit.

## Data Availability

Not applicable.

## Abbreviations

CBO: Community Based Organization
CDA: Critical Discourse Analysis
CSR: Corporate Social Responsibility
ICAP: International Center for Alcohol Policies
IARD: International Alliance for Responsible Drinking
MOA: Memorandum of Understanding
NGO: Nongovernmental Organization
PR: Public Relations
SAO: Social Aspects Organization
WHO: World Health Organization
