# Supplementary material for: Constructing public–private partnerships to undermine the public interest: critical discourse analysis of Working Together published by the International Alliance for Responsible Drinking
Source: Global Health. 2023 Dec 16;19:103. doi: 10.1186/s12992-023-01000-0 (PMC10725627; doi:10.1186/s12992-023-01000-0)
Supplement: Supplementary file 1 — Additional file 1: Fig. S1. Toolkit cover from International Alliance for Responsible Drinking [1] and earlier version from International Centre for Alcohol Policies [2]. Fig. S2. Recurring image in ICAP Toolkit. [file 12992_2023_1000_MOESM1_ESM.pdf]

Additional file 1: Toolkit provenance and presentation

Fig 1. Toolkit cover from *International Alliance for Responsible Drinking* [1] and earlier version from *International Centre for Alcohol Policies* [2]

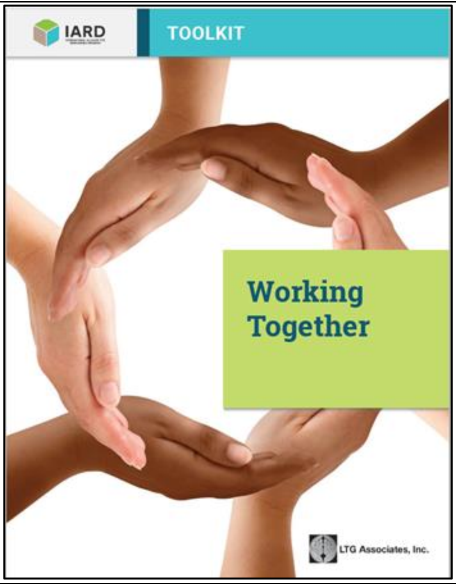

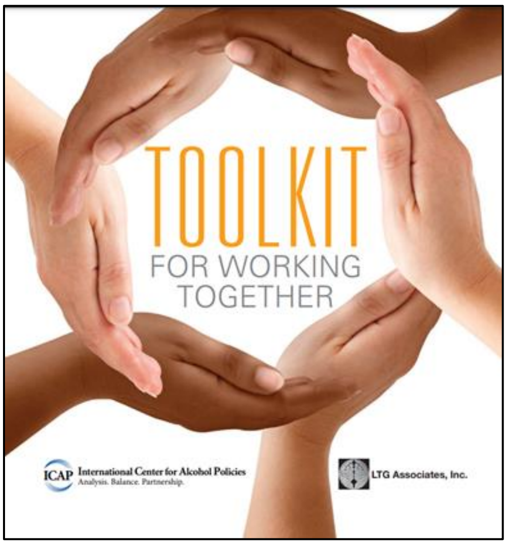

On the front cover of each document is the same colour image of six, slightly cupped, feminine hands with different skin tones. These are arranged, anti-clockwise in an overlapping circular pattern, connoting reaching out, receiving, working together, or cooperating. The image is one of global inclusivity, the world coming together. It is also possible to read the image against the grain, through the lens of "elite capture", a concept which originated in the study of 'developing' countries to describe the way the advantaged tend to gain control over resources meant for others [3], as powerful corporate actors usurping and exploiting a language and imagery originally aimed at including the marginalized and less powerful because of deep inequity in global health.

Each cover also carries the logo of LTG Associates Inc., self-described on its social media account as, "the oldest anthropology based consulting firm in North America" [4]. Other clients of LTG Associates Inc. include health focused organizations including the WHO and in the US, the Robert Wood Johnson Foundation and National Association of County and City Health Officials [5]. There is no introduction of LTG Associates Inc. in the text of the toolkits and no mention of IARD on LTG Associates website, but ICAP was listed as a private client as early as 2013 [6]. This, and the earlier

iteration, suggests that the *Working Together* toolkit was commissioned by ICAP, and the project carried over to IARD. It is clear from the website that projects did carry over, for example, the *Drink Drive Training Workbook* toolkit which was listed on the IARD website on January 13th, 2016 (IARD copyright date 2015), has the file name, "english-ICAP-drink-drive-manual.pdf" [7]. The straightforward continuity between ICAP and IARD is also evidenced in the text of that document, which states: "The development of this training course has been funded by the International Alliance for Responsible Drinking" (p5). Mention of IARD on p6 leads to a footnote listing explicitly *ICAP* sponsoring companies.

The ICAP logo on *Toolkit for working together* has the strapline: "Analysis, Balance, Partnership". The IARD logo on *Working together* has: "Action on Alcohol and Global Health", indicating a shift to a particular focus on global health. The graphics and layout differ slightly and a recurring cropped image of a handshake between a white man and a black woman, the sleeves of their jackets denoting business wear, is not in the IARD version (Figure 2).

Fig 2. Recurring image in ICAP Toolkit

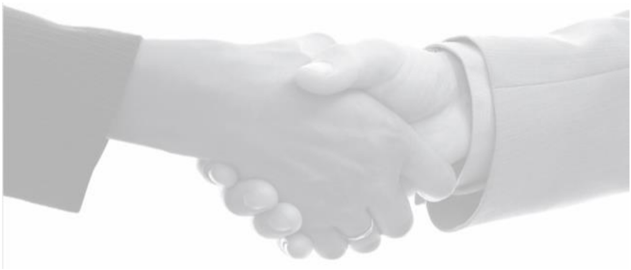

1. IARD. Working Together 2015 [Available from: https://iard.org/getattachment/04c97b59-4cdd-47f8-bd4c-f19ad9d4f38e/tk-working-together.pdf.
2. ICAP. Toolkit for Working Together: International Center for Alcohol Policies; 2012 [Available from: https://web.archive.org/web/20120504022859/http://www.icap.org/PolicyTools/Toolkits/ToolkitforWorkingTogether/tabid/541/Default.aspx.
3. Táíwò O. Elite Capture How the Powerful Took Over Identity Politics (And Everything Else). London: Pluto Press; 2022.
4. LTG Associates. LTG Associates, Inc.LinkedIn 2022 [Available from: https://www.linkedin.com/company/ltg-associates-inc-/about/.
5. Ltd. LA. Projects 2023 [Available from: https://ltgassociates.com/projects/.

6. LTG Associates. Private Clients 2013 [Available from: https://web.archive.org/web/20130206102850/http://www.ltgassociates.com/private.html.
7. IARD. Drink Driving Training Program Workbook 2015 [Available from: https://www.iard.org/getattachment/5fdb32aa-b373-4ede-9584-4f8e48058195/english-icap-drink-drive-manual.pdf.
